# Involving people affected by a rare condition in shaping future genomic research

**DOI:** 10.1186/s40900-021-00256-3

**Published:** 2021-03-15

**Authors:** Jack S. Nunn, Kylie Gwynne, Sarah Gray, Paul Lacaze

**Affiliations:** 1 School of Psychology and Public Health, La Trobe University, Melbourne, VIC Australia; 2 Director of Science for All (Education Charity registered in Australia), Melbourne, Australia; 3 Faculty of Medicine, Health and Human Sciences, Macquarie University, 3/75 Talavera Rd Macquarie University, Sydney, NSW 2113 Australia; 4 President of AusEE Inc (Health Promotion Charity registered in Australia), PO Box 9303, Pacific Paradise, QLD 4564 Australia; 5 Department of Epidemiology and Preventive Medicine, School of Public Health and Preventive Medicine, Monash University, Melbourne, Australia

**Keywords:** Co-design, Rare disease, Online, Participatory, Standardised data on initiatives, COVID-19, Case study, Genomics, Gastrointestinal

## Abstract

**Background:**

There is evidence that involving potential participants and the public in co-designing research can improve the quality of the study design, recruitment and acceptability of the research, but appropriate methodologies for doing this are not always clear. In this study we co-designed a way of involving people affected by a rare genomic disease in shaping future genomic research about the condition. The aim was to report the process, experiences and outcomes of involving people in genomic research in a standardised way, in order to inform future methods of involvement in research co-production.

**Method:**

Participants were recruited from an online community hosted by an Australian-based rare disease charity and were over the age 18 years. Once people gave consent, we shared learning resources with participants and invited them to complete an online survey before joining a two-week facilitated online discussion, followed by a second online survey. We used the novel tool ‘Standardised Data on Initiatives - Alpha Version 0.1’ (STARDIT) to map preferences, plan involvement and report any outcomes from the process, with quantitative data analysed descriptively and qualitative data thematically analysed.

**Results:**

Of the 26 people who gave consent and completed the initial survey, 15 participated in the online discussion and 12 completed the follow-up survey. STARDIT was used to report six outcomes from the process, including 60% of participants’ responses showing a change towards ‘widening’ their view of who should be involved in research to include more people. Outcomes also included an improved understanding of research and how to be involved. Participants enjoyed online discussions, found learning resources useful and asked to stay involved in the research process. The partner organisation reported that a similar online discussion will be used in future research prioritisation processes.

**Conclusion:**

Involving people in co-designing the process improved the study design, ensuring it met the needs of participants. Whilst the study includes participants from only one disease group, using STARDIT allowed us to map people’s preferences and report the methods and outcomes from involving people, providing a way for learning from this case study to inform future research studies beyond the discipline of public health genomics.

**Supplementary Information:**

The online version contains supplementary material available at 10.1186/s40900-021-00256-3.

## Plain English summary

There is evidence that involving potential participants and the public in co-designing research can improve the quality of the study design, recruitment and acceptability of the research, but appropriate methodologies for doing this are not always clear. This mixed methods study examined ways of involving people affected by a rare disease in shaping genomic research.

In this article we describe how people were involved in the co-design process, and ways to plan, report and evaluate involvement in research, including impacts. We demonstrate using a novel way of sharing data in a standardised way to plan, report and evaluate how participant involvement activities positively impacted the study design (Standardised Data on Initiatives - Alpha Version 0.1 - STARDIT). STARDIT is an open access data-sharing platform being developed to standardise the way that information about initiatives is reported.

We conducted pre and post surveys and facilitated online discussions. There were six outcomes from the process, which included participants reporting an improved understanding about how to get involved in research and that learning resources were useful. Participants reported changed views about involvement, with most participants ‘widening’ their perception of who should be involved in research to include more people. Participants enjoyed online discussions and asked to stay involved in the research process. The partner organisation reported that similar online discussions will be used in future research prioritisation processes. Standardised reporting of this study will help inform the future involvement of participants and the public in the design and conduct of genomic research.

## Introduction

Genomic research may lead to better understanding and management of the Eosinophilic Gastrointestinal Disorders (EGID), including Eosinophilic Oesophagitis (EoE). EGIDs are long-term (chronic) inflammatory conditions that affect the lining of the throat, stomach and gut (epithelium). EoE is the most common type of EGID and is most likely caused by exposure to food antigens [1]. EoE affects people of all ages, gender and ancestral backgrounds [2]. Genomic research may lead to better understanding and management of the disease [3]. EoE is a rare disease and involving people affected by EoE in shaping future research could help ensure that the research is relevant, well-designed and aligned with patient priorities [4, 5]. However, currently there is no standardised way of planning and reporting how people are involved in shaping future genomics research [6].

This illustrative case study documents a participatory action research process with the charity AusEE, to co-design a way of partnering with people affected by EoE (including their carers), to help shape future research. The study aimed to examine the processes, experiences, barriers and enablers of participant involvement in genomic research about one condition EoE to inform methods of genomic research co-production. In this article we aim to outline how people were involved in the co-design process, and how Standardised Data on Initiatives (STARDIT) can be used to plan, report and evaluate involvement in research, including impacts [7].

We created a prototype online discussion forum, guided by the principles of participatory action research (PAR) from the International Collaboration for Participatory Health Research, and guidance on co-design [8, 9]. This allowed us to explore and report the practicalities, enablers and barriers of using an online discussion as a way of involving people in genomic research.

Involving the public, patients, research participants and other stakeholders in actively contributing to the research process can lead to a range of positive outcomes. These can include improving the recruitment [10], quality and relevance of research [6, 11]. Involvement is when research is carried out ‘with’ people rather than ‘on’ them [12]. In human genomics research, the need to involve the public and other stakeholders is a crucial aspect of responsible research practice, which can help ensure outcomes of importance to all stakeholders are included in decision making processes [5, 6, 13, 14]. The term ‘stakeholder’ means anyone who has a ‘stake’ in the research, in particular those with important knowledge, experiences, expertise or views that should be taken into account [13, 15]. This can include people affected by Eosinophilic Oesophagitis (EoE), the study team, and the wider public, although in this process we did not involve the wider public. At the earliest stage of the research cycle (the conceptual stage), people affected by EoE were involved in the co-design of this study.

## Methods

In order to co-design the study from an early stage, a representative from AusEE was contacted, inviting the organisation to partner with the research team, with a representative from AusEE invited to be part of the study team and another invited to give feedback on the proposed study design. The representatives were involved in a number of tasks including reviewing and improving the written information, online survey questions, and the facilitation plan for the online discussion.

In order to facilitate comparison with other studies, we used the novel tool ‘Standardised Data on Initiatives - Alpha Version 0.1’ (STARDIT) to map preferences, plan involvement and report any outcomes from the process [7]. This included reporting co-design positively impacted the study. STARDIT is an open access data-sharing platform being developed to standardise the way that information about initiatives is reported across diverse fields and disciplines, including information about which tasks were done by who. Quantitative data was analysed descriptively and STARDIT was also used in parallel with a thematic analysis, which organised data into pre-defined ‘super-categories’ which allow consistent comparison with other data using STARDIT.

Learning resources were both co-created and selected by the investigator team, working in partnership with the Australian Genomics Health Alliance and co-refining the selection with potential participants, including working in partnership with potential participants and the Australian Genomics Health Alliance, using the outcomes of a landscape analysis of educational materials as a starting point for selecting resources [16]. Final resources were checked by a medical professional specialising in EoE.

### Study design

A participatory action research (PAR) paradigm was chosen to guide the process with co-design and reporting informed by guidance from a number of sources [17–19]. PAR is an umbrella term which describes a number of related approaches, including forms of action research which embrace a participatory philosophy and include ‘co-design’ and ‘co-production’ of research [20]. It is a process whereby researchers, the public and other relevant stakeholders “work together, sharing power and responsibility from the start to the end of the project” [21], including knowledge generation and translation [21]. Elements of this study were co-designed in parallel with another similar study, therefore some aspects were inflexible and thus ‘co-refined’ rather than ‘co-designed’.

We used case study methodology to record and describe our experience involving participants in an online discussion about genomics research. The selection of the population for this case study was informed by a number of factors which were appraised by the study team, including ethical, pragmatic and population considerations [22, 23]. One of the investigators (PL) had a professional relationship with the charity AusEE, which was used as a starting point by the study team to explore the appropriateness of the case study.

The case study is presented as an instrumental case study, where the purpose is to understand the particular case and can attempt to provide data that could produce useful generalisations by using inferences from the data [24] (p109). We collected and analysed both qualitative and quantitative data during the involvement activities, informed by a number of frameworks and standards [25, 26].

In addition, we analysed other data from participant survey responses, online discussions, meeting notes, emails, surveys of the study team and reflexive diary entries of one member of study team (JN). Coding and thematic analysis of qualitative data was carried out by two authors independently (JN, KG) and checked by another author (PL), following best practices for enhancing validity in qualitative methods [27]. Two authors of this paper also shared personal comments in the online discussion (KG, SG), which have been anonymised using participant numbers. In order to aid analysis and comparison with other case studies, we used a novel way of sharing data in a standardised way (Standardised Data on Initiatives - Alpha Version 0.1) to map preferences for involvement, plan involvement, report and evaluate how people were involved in the PAR process, and how this positively impacted the study design [7].

STARDIT is an open access data-sharing platform being developed to standardise the way that information about initiatives is reported across diverse fields and disciplines, including information about which tasks were done by who. It also offers a way to add updates throughout the lifetime of an initiative, from planning to evaluation and reporting any impacts. Authors from this paper were involved in co-creating STARDIT, and learning from this process informed the development of the reporting tool [28].

### Participants and recruitment

This study recruited participants from an existing online community managed by the Australian-based charity AusEE. A link to the informed consent form and learning resources was shared by AusEE on a closed Facebook group. We recruited people if they were either a parent, a carer, a partner, family member or loved one of someone with EGID who is under 18; or someone who was over 18 with EGID. If people gave consent, they were invited to complete an online pre-discussion survey and sent instructions for joining the online discussion.

### Stages of research

**Fig. 1:**
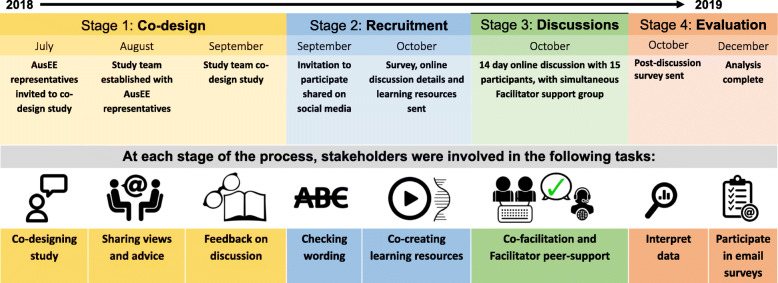
Stages of research

There were four stages of the research process: 1. Co-design; 2. Recruitment and surveys; 3. Online discussions and 4. Evaluation and reflection. The multiple stages of the co-designed research are summarised in ‘Fig. 1: Stages of research’.

Initial contact was made with AusEE in July 2018 and two investigators from AusEE were invited to join the study team (SG, KG). After ethics approval was obtained, an invitation to participate in the study was shared in the AusEE Facebook group and member newsletter in September 2018. This group has members who live in both Australia and New Zealand. Before being asked to give consent, the study was summarised in plain English and two learning resources were shared with participants in order to provide context to the study (see Additional file 1 ‘Data and analysis’).

The invitation to participate contained a link to the participant information document and the informed consent form. If people gave consent to participate, they were invited to complete an online pre-discussion survey, which had demographic data categories informed by other similar studies to allow comparison [29].

**Fig. 2:**
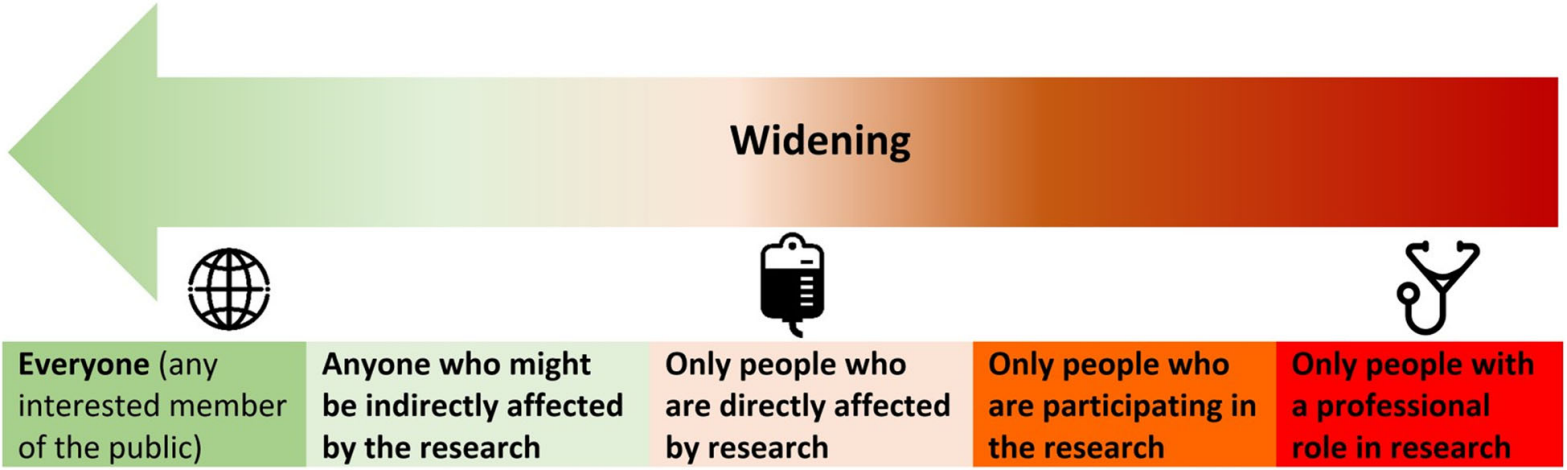
Who should be involved in research?

**Fig. 3:**
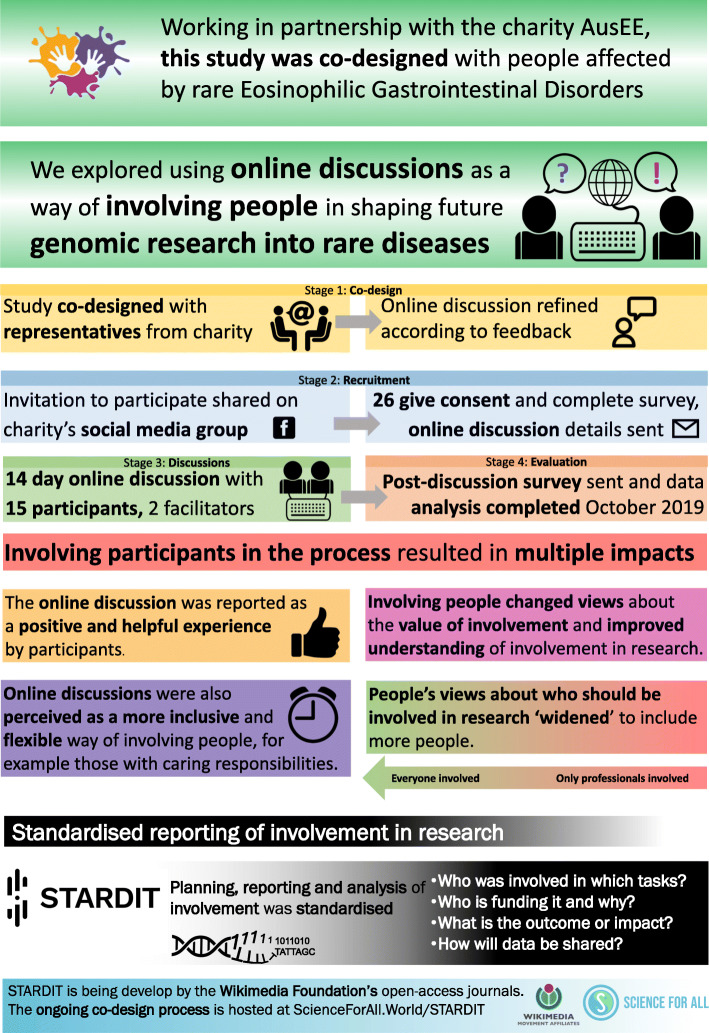
Process summary

Participants who gave consent were then contacted by email, with information about joining the discussion shared alongside relevant learning resources. Participants were also sent a follow-up survey after participating in the online discussion. Questions relating to ‘Who should be involved in research’ were the same as in the pre-discussion survey to allow comparison. Participants could choose from the categories outlined in Fig. 2, with a change in direction towards more people being involved labelled as ‘widening’, the inverse as ‘narrowing’. The entire research process is summarised in Fig. 3.

### Online discussion methodology

We conducted an online discussion to maximise flexibility about when participants could contribute (for example, people in different time-zones or those with different caring responsibilities). A significant advantage of online discussion platforms is that they are accessible to a greater number of people. This creates a more equitable platform for people to participate, compared to a synchronous (simultaneous ‘real-time’) discussion.

We offered anonymity in online discussions to avoid people inadvertently disclosing sensitive or personal information, which may have serious implications, for example, impacting on eligibility for future health insurance. Participants could choose to use their real name and email address, or remain anonymous by using pseudonyms or temporary email accounts.

Before participants joined the online discussion, seven learning resources were shared with them. This included a short 60 s online video about the study context and purpose [30], a one-page infographic summary of a scoping review about genomics research [31], and a short two-page summary of genomics and contemporary research relating to EoE was co-created with AusEE, the study team and experts in genomics (see Additional file 1 ‘Data and analysis’) [32].

An open-source software web application ‘Loomio’ [33] was installed on virtual machines hosted by an Australian Government initiative called ‘Nectar Cloud’ [34]. Participants could securely log-in from anywhere in world and participants’ data was stored securely on servers physically located inside Australia.

Two people facilitated and moderated the discussion for 14 days (JN, KG), one of whom was a parent of a person affected by EoE (KG). Participants were invited to co-create their own boundaries for the group discussion at the start by reviewing provided statements and suggesting amendments. The facilitators judged when to introduce new topics (depending on the engagement with each topic) with the recommended schedule below in Table 1 used as a template.

**Table 1:** Online discussion overview

Question	Day
What do you understand by the word ‘research’?	Day 1
What do you understand by the phrase ‘genomic research’?	Day 1
Why do we do research?	Day 2
Which aspects of any future genomic research should be influenced by the following groups of people?	Day 4
What methods do you think could be used to involve those people in future genomics research?	Day 6
Do you have any ideas, thoughts or reflections that have not been shared yet?	Day 7
Discussion closed	Day 14

## Results

### Stage 1: co-design

The input of the representatives during the planning and co-design stage had clear positive impacts, particularly in improving educational resources and ensuring the online discussion was advertised using wording appropriate to the existing online community. For example, representatives from AusEE helped change the study design to include explicit opportunities for participants to learn more about genomics and EoE, avoiding participation being perceived as having a one-way benefit. During the co-design process it was also decided to exclude people who were under 18 and people who stated they were representing someone who was over 18, as people who were 18 and over had the choice to represent themselves.

### Stage 2: recruitment and surveys

In total 26 participants completed the pre-discussion survey, 12 completed the follow-up survey. These responses are summarised in Table 2. All but one of the participants were female, with most reporting they were parents of a person with an EGID. Self-reported educational attainment was mostly ‘degree (bachelors), diploma or post-graduate’, with one participant reporting they had professional experience in genomics. Most participants were between 30 and 45 years old and participants all lived in Australia, except for one who lived in New Zealand.

**Table 2:** Summary of pre and post survey responses

Question	Results
What made you decide to respond to our invitation to participate in this project? 22 responses (pre-discussion)	Fourteen participants stated they decided to participate as they wanted to help improve knowledge of the disease and help find a cure. Two people were specifically interested in genomic research [P6, P9], and one person reported they were ‘researching themselves’ and their sons’ genomic variations [P5].
What do you hope to get out of participating in this discussion? Do you have any specific expectations? 20 responses (pre-discussion)	Four participants wanted to ‘learn more’ [P16]. Five participants stated they wanted better outcomes for patient care and treatment protocols, with three participants stating they wanted to be actively involved in helping research to improve outcomes. Two participants wanted to hear the perspectives of others. Five participants stated they had no expectations.
Do you have any ideas about how the different people could influence future research? 19 responses (pre-discussion)	One participant stated that people with a rare disease and their families are ‘likely to have different priorities from scientists’ [P16]. Another suggested that ‘sharing patient experiences, priorities of research areas’ and involving people in co-defining ‘ultimate patient outcomes’ were ways people could influence research [P21].
Is there anything in particular you liked or thought was helpful about how the discussion was conducted? 8 responses (post-discussion)	One participant said they ‘enjoyed the interaction, helpful links with information about genomics and the topic threads’ [P21]. Another added they liked ‘being able to read others thought processes on each topic’ [P3]. Others said ‘the responses to mine were timely and provoked further questions that made me think in new directions’ [P25]. Two participants said that ‘having different topic questions/threads was helpful’, as was the ‘information provided to start’ of some threads [P29]. One participant added that ‘it was great because even though busy with my son’ they could travel and ‘still catch up and learn things and do my input too’[P30].
Is there anything you didn’t like, thought was unhelpful or could have been improved about how the discussion was conducted? 4 responses (post-discussion)	Only two participants provided answer for this question other than ‘no’. One participant stated ‘I didn’t like the platform it was conducted on’ as it was not ‘user-friendly’ [P15]. Another added that sometimes facilitators ‘added another question too quickly before a number of people had a chance to answer the first one’ which risked leaving ‘some people behind’ [P3].
Did you have any expectations from participating in this research that were met or not met? 8 responses (post-discussion)	One participant stated that they wanted to ‘learn more about the difference of the gene and genomics and that was met’ [P30], and three others stated their expectations had been met, with none reporting they had not.

A total of 41 responses were given by 12 participants who shared their identity at both the baseline and follow-up, including ten questions about which aspects of genomic research should be influenced by different stakeholders which identical at each stage. Of the 41 responses to the ten questions, 60% showed a change towards ‘widening’ involvement (*N* = 25/41), 36% of responses stayed the same (*N* = 15/41) and 7% ‘narrowed’ (*N* = 3/41). Recurring themes were identified by the study team, and six specific outcomes were reported as a result of involving participants in this process.

### Stage 3: online discussions

**Fig. 4:**
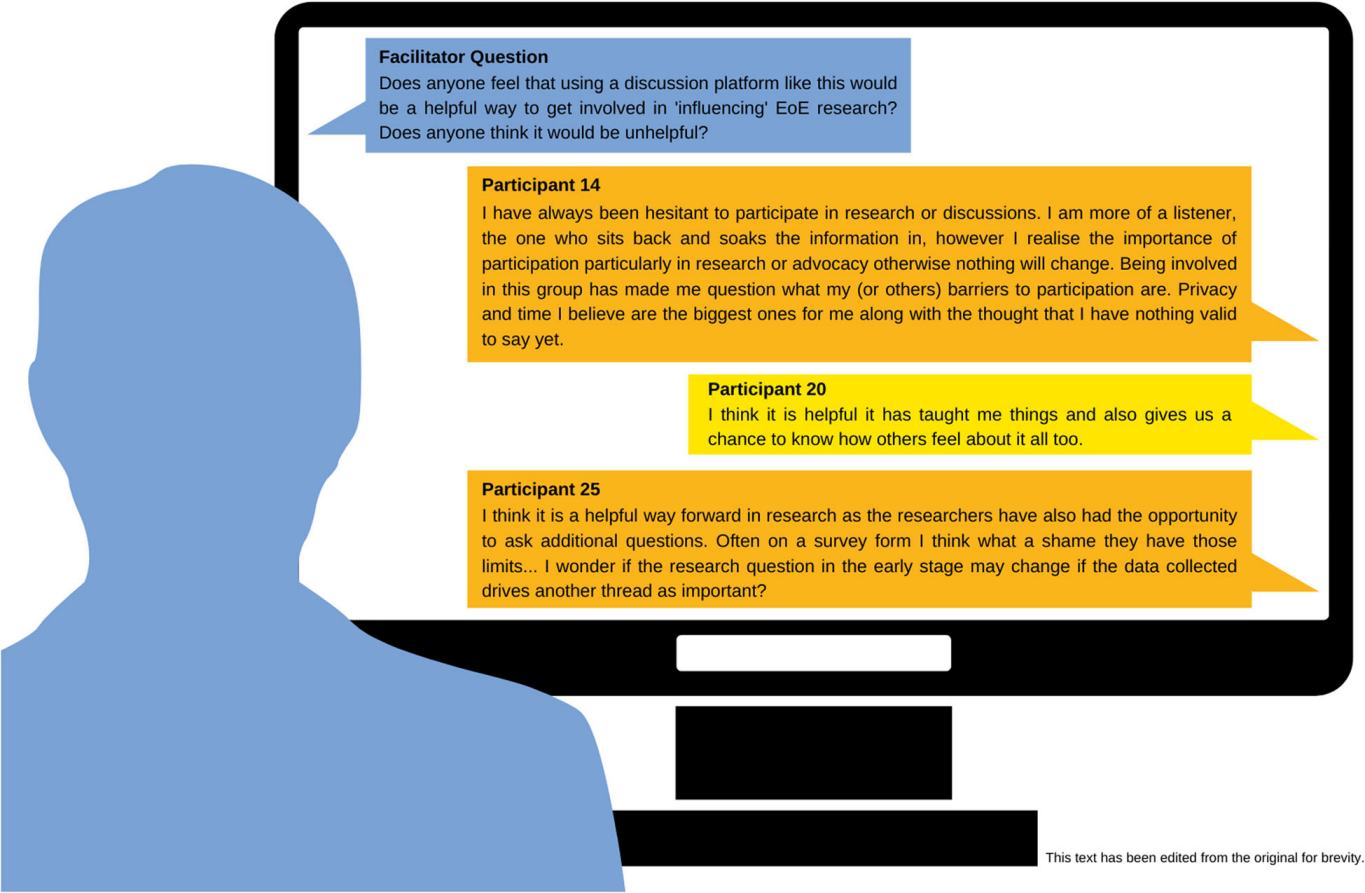
Online discussion visualisation

A total of 15 people participated in the online discussion. The President of the charity AusEE also identified herself by name in the discussion (SG). All but two participants chose to use their real name in the online discussion, which they had provided when registering and giving consent. A visual representation of the online discussion is illustrated in Fig. 4.

### Stage 4: evaluation and reflection

Participant feedback about the online discussion was sought via survey and was positive, with one participant describing the process as ‘very good’ [P25]. Participants reported that they enjoyed being involved and that participating in the discussion changed their views about involvement. The visual summary of the scoping review about involvement in genomics that was shared at the start of the discussion was described as a ‘great summary document’ [P21]. One facilitator noted the group were ‘well informed’ about research [P16], with the discussion indicating a high level of general understanding about research, genomics and the associated ethical, legal and social implications. For example, one participant stated ‘I wonder about the limitations of genomic sequencing but believe knowing the result is an adult human right’ while adding they were unsure ‘how much’ they would want to know themselves [P25]. Another participant mentioned the ‘moral minefield’ of pre-implantation genomic screening, noting that “not all that is ‘marked’ will come to be” [sic] [P3].

### Outcomes from the process

There were six outcomes reported from this process, which are summarised in Table 3.

**Table 3:** Summary of outcomes from the process

Outcome	Summary
1. Learning resources useful	Participants stated that the learning resources were useful and helped improve their understanding of genomics, research and the associated ethical, legal and social implications.
2. Changed views	Participants reported that the process of being part of an online discussion gave them an opportunity to ‘learn a lot’ about the views and experiences of others and reflect [P25]. Participants reported that this ‘challenged’ them into ‘rethinking’ or changing or their own views on certain issues [P25].
3. Enjoyed online discussions	Four participants said they enjoyed being part of the process and preferred it when compared with other methods such as face to face discussion or interviews.
4. Online discussions to be used in future research prioritisation	The President of AusEE stated that the discussion had ‘given her an idea’ and that she would combine face to face and online discussions into a process to involve people in deciding how the medical research fund is spent.
5. Participants asked to stay involved in the research	Participants requested to stay involved in the research process, including in analysing data and being co-authors on the paper.
6. Improved understanding of how to get involved in research	Participants reported improved understanding of genomics research, including how they could be involved.

### Participant views about involvement genomic research

While the focus of the online discussion was involvement in genomics research, the themes of the discussion reflected the interconnected nature of the subject, including ethical, legal and social implications of genomics research. Issues discussed included research prioritisation and funding; data sharing; health technology assessment, and health and life insurance. Fourteen participants shared the view that they wanted to be involved in improving knowledge of EGID and helping find a cure ‘so other parents don’t go through what we went through’ [P25]. Similarly, participants wanted to help genomic research in the area improve the lives of people affected by an EGID.

Ten participants identified specific enablers of involvement in genomic research, including ‘equity’. Open-source data and information was identified as an enabler of involvement [P3]. Emotional connection to an issue was identified as being either an enabler or a barrier to involvement, with one participant stating ‘there needs to be a balance’ [P21]. Other enablers included participants being ‘able to contribute without putting their personal situation at risk’ with data ‘well protected so as not to be used against an individual or group of people’ [P3].

The theme of the ‘intention’ and ‘purpose’ of research emerged, with a discussion on vested interests. One participant identified that people affected by EGID ‘certainly do’ have a vested interest [P3]. Participants were concerned that research priorities were driven by money. Four participants mentioned ‘insurance’ as an area of concern which requires public involvement and scrutiny with regards to data sharing, with one stating it is a ‘valid community fear’ that research data will be used ‘for’ insurance companies or other for-profit organisations [P3]. One participant stated that ‘progressive watering down of privacy protections’ meant they were concerned their children would be ‘refused insurance because of a decision I have made’[P16], such as participating in research.

### Stakeholder views about the online discussion

Participants identified specific things about the online discussion which they felt enabled participation or were barriers to participating. Participants ‘enjoyed the interaction’ of the online discussion and were ‘supportive, positive and open’ [P21] [P3]. Four participants found the links to learning resources helpful. Participants felt online discussions are ‘good to help’ people get involved, in particular those who are unable to travel or live in remote areas [P20]. The research method used in this online discussion was described as ‘very good’ as the time frame allowed for ‘life responsibilities and also gave time to process new information and think about others’ comments’ [P25]. However, one participant and one facilitator felt the discussion was ‘too quick’ [P3]. Other participants reported the length and pace was good, highlighting that co-design is an important element in designing similar future discussions. One participant stated that ‘this style of discussion here has been interesting and by learning from others I have found some of my initial thoughts have changed’ [P25]. Other participants liked the idea of a multi-stage way of involving people using a combination of face to face and online discussion [P25], which aligns with other models used in priority setting [35, 36].

One participant mentioned that ‘one of the things that I really love about this research is that the research topic is on involving people in research and this research itself does exactly that - involves people in the research’[P21]. The ‘open discussion’ at the end where participants could raise any subject or question was also highlighted as a ‘helpful way forward in research’ as it allows participants to identify and focus on important areas, and researchers an opportunity to ask clarifying questions, additional questions and re-focus larger research questions. Other participants noted they might be influenced by the answers of others and that as this is part of the research process, it would need to be a consideration in data collection methodology [P21]. For example, people might share different information in an anonymous survey compared to an online discussion, owing to concerns about privacy or because another participant has said something which has prompted a memory they might otherwise have forgotten.

## Discussion

Involving participants in co-designing the research process resulted in changes to the study design, including improving language used in recruitment and learning resources. Involving people in online discussions about involvement in research changes people’s views about who should be involved in research, including participants ‘widening’ their views about who should be involved in research to include more people.

Most participants did not seek anonymity during online discussions, and this may reflect that participants were recruited from a social media group where their identities were already known and a sense of community already developed. It may also suggest that participants wished to be known to other participants in order to give and receive support inside and outside of the discussion.

However, optional anonymity in online discussions can also be viewed as an advantage by some participants, by removing elements of identity which may be associated with power disparity, such as appearance, age or gender or future discrimination by health insurance providerr [37]. Anonymity for some may therefore lead to a disinhibition effect, with participants feeling more able to express views and perspectives that they may not feel confident sharing in other contexts.

### Learning from the outcomes

The participants were highly engaged in the process, possibly as all self-reported a personal motivation for improving research in this area. The participants enjoyed being part of the process and none reported difficulties with the platform, which may be a result of familiarity with other online platforms, which may be correlated with self-reported higher degree level education. One participant wrote ‘Thanks for this platform, process, everything really I’ve enjoyed being part of it’ [P21].

However, one participant said that while diagrams and visual aids were helpful, they wanted ‘more diagrams when reading through concepts or ideas’ [P3]. This suggests that online text-based discussions might not suit those with a preference for visual communication and highlights the importance of co-designing online discussions with potential participants who may have diverse learning or access needs.

The learning from ‘Outcome 2: Changed views’ aligns with other studies, showing that the act of being involved in a discussion or in research can have outcomes which are a form of ‘transformative learning’ for both participants and study team members [38]. At the core of the participatory action research method is ‘critical reflexivity’, a process which asks people involved to reflect on the causes of problems, any solutions and the actions that people can take to improve the current situation [8]. It is a form of collective self-reflective enquiry undertaken by people in order to understand their situation from multiple perspectives [39]. In this sense changed views of both researchers and participants involved can be viewed as an impact of ‘transformative learning’, with the reflexive re-examination of views and perspectives part of the PAR paradigm.

### Strengths

While this study was planned and completed before the COVID-19 pandemic, the methods of involving people online described here now have an unexpected relevance to many disciplines, as research projects around the world seek to involve people online in novel ways, and evaluate such methods in a standard way.

Using STARDIT for standardised preference mapping, planning and reporting of involvement meant outcomes from the process could be mapped more effectively [7, 8], including outcomes from this process beyond the date of this publication.

Measuring outcomes such as ‘transformative learning’ can be challenging. This process overcame such challenges by using baseline and follow-up surveys.

It is significant that some participants preferred the online discussion method over face to face discussion or interviews, as this highlights the importance of using STARDIT-PM to map the potential participant’s preferences when co-designing involvement, as this helped ensure research design meets the needs of those participating and those involved. Authors from this paper were involved in co-designing STARDIT, and learning from this process informed STARDIT Beta Version 0.2.

In order to share power effectively and ensure this article reflected everyone’s experience of the process, the two people affected by EGID who were participant representatives were invited to be co-authors of this article. As participant representatives, they were involved from the very earliest stages of this research project. In addition there were opportunities for other participants to give feedback on the manuscript (including checking data analysis). In order to demonstrate the value of participant representatives’ contributions, the study team ensured they were invited to propose their own flexible time frames to contribute effectively to the study. This flexibility, and flexible deadlines from the article publisher in response to COVID-19, helped ensure they could balance providing feedback on data analysis and article versions with any caring responsibilities.

### Limitations

The study design allowed people to be anonymous at each stage, so there was limited data from people who shared identifying information at each stage, meaning that some anonymous participants might be counted twice. However, as this study is a relatively small sample, statistical extrapolation from this case study is limited. This study also recruited from an existing online community which used a social media group, so data may reflect the views of people who have more experience using online platforms than the general public and are thus more competent.

Some participants demonstrated passive behaviour in online discussions. For example, some participants logged-in multiple times, read comments but did not contribute comments. While the follow-up survey attempted to capture views of these participants, it was not completed by everyone, so it is hard to assess why certain people did not contribute more. However, one participant reported learning while reading and not contributing, and therefore there may be under-reporting of some learning outcomes from participants who were more passive [40].

This study was designed in parallel with another similar study to allow comparison of data sets [41], so some aspects were inflexible (such as the choice of open-source discussion platform) and could only be ‘co-refined’ rather than ‘co-designed’.

The decision to host the discussion on a separate platform (Loomio) from the one hosted by AusEE (on Facebook) was made for ethical reasons, as the study team could ensure control and ownership of the data. This may have been a barrier to some who decided not to participate, perhaps as the new platform was unfamiliar. However, data on this was not collected. While the gender balance of the participants was reflective of the wider online community from which participants were recruited at the time, data on educational background was not available so it is unclear if the educational background of participants was statistically representative. Future studies should seek to explore ways of appraising who might be excluded from research using more standardised processes [42].

## Conclusions

This study provides valuable insights to the involvement of participants in research co-design and its associated benefits for researchers and participants. The co-design process improved the design of the study and ensured it met the needs of participants. Through sharing and discussing views in an asynchronous online discussion, participants reported their views changed. This aligns with other studies and demonstrates that transformational learning occurs through the process of involving people. After participating in the online discussion, people’s views about who should be involved ‘widened’ to include more people. Participants wanted to be involved in shaping future genomic research in order to improve it, especially being involved in ethical oversight and scrutiny to improve safeguards regarding data use and privacy.

Whilst the study includes participants from only one disease group, using a standardised reporting tool allowed us to map people’s preferences and report the methods and outcomes from involving people. Such reporting also provided a way to report the benefits to both participants and researchers, providing a way for learning from this case study to inform future research studies beyond the discipline of public health genomics.

## Supplementary Information

**Additional file 1.** Data and Analysis.

**Additional file 2.** STARDIT report.

**Additional file 3.** GRIPP2 report.

## Data Availability

All relevant data has been anonymised and shared in the additional files. La Trobe University is storing all raw data according to the relevant ethics policies, and invites requests for more detailed data. The Wikidata version of the STARDIT report  is available at https://www.wikidata.org/wiki/Q100403236

## Abbreviations

EGID: Eosinophilic Gastrointestinal Disorders
EoE: Eosinophilic Oesophagitis
STARDIT: Standardised Data on Initiatives
PAR: Participatory action research
