# Supplementary material for: Involving people affected by a rare condition in shaping future genomic research
Source: Res Involv Engagem. 2021 Mar 15;7:14. doi: 10.1186/s40900-021-00256-3 (PMC7958104; doi:10.1186/s40900-021-00256-3)
Supplement: Supplementary file 1 — Additional file 1. Data and Analysis. [file 40900_2021_256_MOESM1_ESM.pdf]

Data and Analysis

Involving people affected by a rare genetic disorder in shaping future research

About this document

This document contains additional data relevant to the case study 'Involving people affected by a rare genetic disorder in shaping future research'. In addition it contains a more detailed description of the data sources in this case study. This document includes the preferences mapping data (STARDIT-PM), and other data about this initiative[1]. The corresponding Standardised Data on Initiatives Alpha Version (STARDIT) of the report can be found in 'Additional File 2 - STARDIT report'.

Contents

About this document 1
Survey questions 2
- Pre-discussion survey questions 2
- Post discussion survey questions 5
- Facilitator survey questions 8

STARDIT Preference Mapping (STARDIT-PM) 9
Data 13
- Demographic information 14
- Widening and narrowing for each question asked in pre and post survey 15
- Participant experience 16

Learning resources 16
- Summary of learning resources 17

References 19

Survey questions

Pre-discussion survey questions

After participants had read the Participant Information and given consent to participate, they were asked to complete the following information. The questions below are worded exactly as participants read them.

1. Full Name (if you would prefer to not use your real name you may use a pseudonym)
2. Email address (Please note this needs to be a working email address. If you do not have one leave this blank and we will contact you by your preferred method of communication.)
3. Phone number (optional)
4. Any other preferred method of communication? (Please share any other preferred method of communication if email or phone are not preferred)
5. Age
6. Gender (Choose from ‘Male’, ‘Female’, ‘Transgender’, ‘Intersex’, ‘Other’, ‘Prefer not to say’)
7. Educational background (tick all that apply)
    a. Middle school qualifications (up to age 16) ('lower')
    b. High school qualifications (ages 16-19) (‘middle’)
    c. Degree (bachelors), diploma or post-graduate ('higher')
    d. I have qualifications or professional experience in genomics (professional)
    e. Prefer not to say
8. How would you describe yourself? (Please tick all that apply)
    a. A person with EGID
    b. A parent of a person with EGID
    c. A carer of a person with EGID
    d. A partner, family member or loved one of a person with EGID
    e. Other (please describe)
    f. Prefer not to say
9. In which country do you live (or spend most time)?
10. Please tick which statement applies
    a. I am a parent, a carer, a partner, family member or loved one of someone with EGID who is under 18

b. I am a parent, a carer, a partner, family member or loved one of someone with EGID who is 18+
c. I am over 18 and am representing myself

11. What made you decide to respond to our invitation to participate in this project?
12. What do you hope to get out of participating in this discussion? Do you have any specific expectations?
13. There are many benefits of involving people other than researchers in the co-design of research studies at every stage of the research cycle. Research suggests that involving people improves the quality and the relevance of the research. Involving people can also improve participant experience and increase participation. **Who do you think should influence what kind of genomic research should be done in the future?**
14. What makes you say that? (why did you give that answer?)
15. Do you have any ideas about how the people from your previous answer could influence future research? (For example, what tasks could people affected by EGID be involved in?)
16. **Which aspects of any future research genomic research should be influenced by the following (**participants were presented with a grid of tick boxes, the horizontal axis being who should be involved, the vertical a list of tasks. The horizontal was as follows)

Everyone (any member of the public who is interested)	Anyone who might be indirectly affected by the research	Only people who are directly affected by the research	Only people who are participating in the research	Only people with a professional role in research

a. All aspects mentioned below (leave others blank if ticking this)
b. Finding questions to ask (identifying research topics)
c. Deciding which questions to prioritize and fund
d. Deciding how to try and answer the question (the research method)
e. Attempting to answer the question (carrying out the research, including collecting information)
f. Trying to understand if it is possible to answer the question (analyzing the information)
g. Sharing the information that has been found, and any answers that may have emerged (dissemination and publication)

   h. Ensuring that any information or answers are able to be used to help people in practice, policy or future research (sometimes called research translation)
   i. Deciding if the way of asking the question and all the other stages of the research were appropriate (evaluating the research method and any impacts)
   j. Designing how people are involved in the research

17. Have you ever participated in research in the past? (by participation, we mean as a research subject – for example part of a trial)
    a. Yes
    b. No
    c. Prefer not to say
    d. Unsure
18. Have you ever participated in research in the past? (by participation, we mean as a research subject – for example part of a trial)
    a. Yes
    b. No
    c. Prefer not to say
    d. Unsure

Post discussion survey questions

1. How would you rate the following? (chose from ‘Excellent’, ‘Somewhat good’, ‘Neither good nor bad’, ‘Somewhat poor’, ‘Extremely poor’)
    a. Your overall experience of participating in the online discussion
    b. Your assessment of how we conducted the survey and discussion format
    c. The support you received to be involved (for example, practical support such as instructions for using the online tools)?
    d. Information and learning materials you were given before the event
2. Did you feel you meaningfully contributed to the discussion?
    a. Yes
    b. No
    c. Unsure
3. Is there anything in particular you liked or thought was helpful about how the discussion was conducted?
4. Is there anything you didn’t like, thought was unhelpful or could have been improved about how the discussion was conducted?
5. Do you have any other thoughts, ideas or comments?
6. Would you like to be updated about the progress of the research and offered chances to be involved where possible? (Chose ‘yes’ or ‘no’)
7. Did you have any expectations from participating in this research that were met or not met?
8. Have any of your views and perspectives about involving people in genomic research changed since participating in this research? If so, please describe.
9. There are many benefits of involving people other than researchers in the co-design of research studies at every stage of the research cycle.
10. Research suggests that involving people improves the quality and the relevance of the research. Involving people can also improve participant experience and increase participation. **Who do you think should influence what kind of genomic research should be done in the future?**
11. What makes you say that? (why did you give that answer?)

12. Do you have any ideas about how the people from your previous answer could influence future research? For example, what tasks could people affected by EGID be involved in?
13. **Which aspects of any future research genomic research should be influenced by the following (**participants were presented with a grid of tick boxes, the horizontal axis being who should be involved, the vertical a list of tasks. The horizontal was as follows)

Everyone (any member of the public who is interested)	Anyone who might be indirectly affected by the research	Only people who are directly affected by the research	Only people who are participating in the research	Only people with a professional role in research

    a. All aspects mentioned below (leave others blank if ticking this)
    b. Finding questions to ask (identifying research topics)
    c. Deciding which questions to prioritize and fund
    d. Deciding how to try and answer the question (the research method)
    e. Attempting to answer the question (carrying out the research, including collecting information)
    f. Trying to understand if it is possible to answer the question (analyzing the information)
    g. Sharing the information that has been found, and any answers that may have emerged (dissemination and publication)
    h. Ensuring that any information or answers are able to be used to help people in practice, policy or future research (sometimes called research translation)
    i. Deciding if the way of asking the question and all the other stages of the research were appropriate (evaluating the research method and any impacts)
    j. Designing how people are involved in the research
14. Full Name (Optional- if you would prefer to not use your real name you may use a pseudonym)
15. Email address (optional)
16. Phone number (optional)
17. Age
18. Gender (Choose from ‘Male’, ‘Female’, ‘Transgender’, ‘Intersex’, ‘Other’, ‘Prefer not to say’)

19. Educational background (tick all that apply)

a. Middle school qualifications (up to age 16) ('lower')
b. High school qualifications (ages 16-19) (‘middle’)
c. Degree (bachelors), diploma or post-graduate ('higher')
d. I have qualifications or professional experience in genomics (professional)
e. Prefer not to say

20. How would you describe yourself? (Please tick all that apply)

a. A person with EGID
b. A parent of a person with EGID
c. A carer of a person with EGID
d. A partner, family member or loved one of a person with EGID
e. Other (please describe)
f. Prefer not to say

21. In which country do you live (or spend most time)?

Facilitator survey questions

1. Please describe your tasks in the process of involving people in planning of the study
2. What did you learn from the process of involving participants in the research planning phase?
3. Please describe specifically what worked well or was useful about the way the study was conducted (including how people were involved)
4. Please describe specifically what did not work well or was not useful about the way the study was conducted (including how people were involved)
5. Were there any barriers or enablers to conducting the study or involvement activities? (institutional or otherwise)
6. Do you think the involvement activity achieved its intended aim(s)?
7. Do you think the study achieved its intended aim(s)?
8. Do you have any advice to other researchers planning involvement for their research?
9. Do you have any advice to other researchers planning to involve people using online discussions?
10. Describe the impact you think involving people had (positive/negative - on the research, staff or participants)
11. Who do you think should influence the kind of human genomic research done in the future, and why? (e.g. the public, participants of research studies, doctors, school children, politicians etc)
12. Which stages of future genomic research should be influenced by people other than researchers (if any)? (e.g. concept planning of new studies, study design, conducting the research, presenting the results etc)
13. Other comments

STARDIT Preference Mapping (STARDIT-PM)

This table uses the Alpha version of the Standardised Data on Initiatives Preference Mapping (STARDIT-PM) to categorise the data into certain areas [1]. Preferences were recorded from all data sources, including the initial survey, online discussion with participants, online Facilitator discussions, follow-up surveys with participants and with Facilitators. Facilitator comments were only included from one Facilitator with personal experience (KG), and comments from the other Facilitator were excluded from analysis (JN). If the same participant made the same point at different stages, this was counted as one view. The standardised categorisation is intended to facilitate comparison with other studies. Accordingly, the content may be similar to other sections of the qualitative thematic analysis.

STARDIT-PM area and quantitative data	Qualitative summary
Views on who should be involved:<br><br>**17 participants** shared views about who should be involved	One participant wrote that asking ‘Who should be ‘excluded’ is a helpful starting point when answering this question’ [P3]. Participants contributed 17 different statements saying patients and their families should be involved. The word ‘collaboration’ was used to describe how people should work together. Besides patients and families and those ‘directly affected’ [P3], other ‘specialists’ and groups were mentioned by participants [P23]. These included including doctors, medical professionals, researchers, patient advocacy groups, immunologists, gastroenterologists and IT experts. One participant stated ‘drug companies will have a part to play’ and went on to say ‘this would need supervision and strict guidelines’ [P9].
Views on specific tasks people involved could do:<br><br>**10 participants** shared views about specific tasks people involved could do	Participants shared multiple tasks which they felt patients should be involved in. Identifying topics and ‘what’s a priority’ was mentioned 5 times [P3]. One participant wrote ‘medical researchers should take the opportunity to be guided more from the patient themselves on an idea/direction for a research project rather than the other way round’ [P21]. Involving people affected in setting outcomes was also mentioned by participants, including being involved in saying what ‘would be useful’ to patients [P22]. Involving people affected ‘at the design stage’ of research was mentioned, with two participants mentioning surveys as an example. For example, a ‘quality of life survey’ was mentioned as a way people could make sure the right questions were being asked [P21]<br>[P25]. Fundraising, campaigning and ‘advocating’ were mentioned as ways of ‘raising awareness’ by two participants [P5] [P9]. Being involved in ‘doing’ research was mentioned by one participant [P3].<br>Being involved in working with insurance companies and government to explore the legal, financial and privacy impacts of how a diagnosis might affect people was mentioned by one participant [P3]. One participant mentioned involving patients in discussions about health technology assessment, including ‘health economics and the consequences’[P3].
Views on modes of communication:	Two participants mentioned websites, with ‘public government research websites’ and ‘patient advocacy groups’ as ways of advertising opportunities for involvement or participation. Face to face research

**2 participants** shared views about preferred communication modes	and online surveys were also mentioned as other modes. Online discussion, face to face communication were also mentioned.
Views on what methods should be used to involve people:<br><br>**6 participants** shared views about what methods should be used to involve people	One participant mentioned **surveys**, stating that 'short surveys' completed on computers or phones are 'easy' and cost effective, especially while people 'wait for their medical appointment' [P25]. Groups such as '**volunteer committees'** were suggested as a method. One participant suggested that public awareness 'facilitates involvement' [P25] and that **people sharing stories** can be a source of 'support or inspiration', in particular 'public figures' [P25]. **Online and face to face support groups** were identified as an area for 'uncovering trends', the first stage in identifying research topics [P3] 'Community discussions' [P16] and 'working collaboratively' with professors and 'those directly affected' [P3] was mentioned by two participants. Similarly one participant stated 'patient advocacy groups partnering with researchers is valuable for all involved' [P28]. One participant stated she liked 'focus groups as face to face' as when working in an online group the 'intent of the writer' can be interpreted differently to as it was intended (for example, presuming an incorrect tone of voice)' [P21]. Another participant preferred a combination of face to face events once or twice a year with most work being done by teleconferencing, owing to geographical separation [P3]. Online video teleconferencing was also suggested as a good method as participants 'get a much better feel for people and their thoughts because you can see them (get all the cues), then break away for specific follow-up' [P3]. Another participant agreed a 'two stage' approach would be good, starting with involving people online, then meeting face to face [P25]. Ensuring online discussions have a mixture of both open and closed questions allows the open questions to 'create another idea' and novel discussion [P3].
Views on facilitators of involvement:<br><br>**10 participants** and **3 Facilitators** shared views about facilitators of involvement	One participant noted that 'Participants need to be able to contribute without putting their personal situation at risk'. One participated noted that 'more respect for the patient and their family, their knowledge & experience' would facilitate involvement. Two participants noted 'Power and knowledge' as facilitators [P20], highlighting not having 'payment for access' to information as a facilitator [P3]. Emotional connection to an issue can be both a facilitator and a barrier to involvement, with a 'balance' highlighted as a facilitator of involvement [P21]. For those who are unable to travel or live in remote areas, online discussions are 'good to help' people get involved, with one participant stating 'It is hard for us to help with research' if it is not in their area and they are 'unable to afford to travel' [P20]. Good facilitation of face to face events was highlighted by one participant as face to face discussions 'can be endless so need to be governed well' [P3]. Public awareness facilitates involvement, especially stories from people affected. This also can be a source of 'support or inspiration' for some people [P25].

	Six participants reported specific things about the way this study was conducted that facilitated their involvement. One participant ‘enjoyed the interaction’ and four found it ‘helpful’ to have ‘**links with information about genomics’** relevant to the topic threads’ to ‘explain a concept’ [P21, P3, P29, P28]. One participant also stated ‘**being able to read others thought processes** on each topic’ helped [P3]. Another responded that responses in the **discussion ‘provoked further questions that made me think in new directions’** [P25]. Another participant added that other **participants were ‘supportive, positive and open’** [P3]. One participant noted that the **flexibility of being able to join at any time** ‘was great’ as she was often ‘busy caring for her son with EoE and travelling’. They stated that the way the discussion was hosted meant that they ‘could still catch up and learn things’ and also provide input [P30]. Two participants mentioned having **different topics threads with a lead question was helpful** and a ‘good format’ [P29] [P28]. One participant stated ‘This process has been really interesting because we can only write so much, get **a variety of input which makes us think’** [P3].<br><br>Facilitators reported that they ‘needed more support and advice than expected’, stating that the training and ongoing support given before and during facilitation was essential. One Facilitator reported that ‘touching base’ and learning the experiences of other Facilitators and feeling ‘part of the team’ all facilitated their facilitation of online discussion.
Views on barriers of involvement:<br><br>**5 participants** and **3 Facilitators** shared views about barriers of involvement	One participant stated barriers included ‘**payment for access’ to information,** such as paywalls for peer-reviewed information [P3]. Researchers promising ‘**feedback that never came’** was considered a barrier to involvement by one participant [P25]. One participant stated that barriers included **representatives being ‘undervalued and under-supported**.’ [P3]. One participant noted that issues which have been identified should be addressed by ‘the Ministry’ and as a result of inaction, volunteers were ‘working on extra time’. [P3]. **Online discussions ‘can be viewed differently to the intent of the writer’** [P21]. One participant highlighted that **privacy was an issue when involving people** in research using focus groups online or face-to-face. They stated ‘**some people might share more if they didn't have a broader audience - especially people they don't know’**. [P21]. Two participants reported specific things about the way this study was conducted that were barriers to their involvement. One participant reported that although they like the format of the discussion, they ‘**didn't like the platform** it was conducted on’ as it was not ‘user friendly’ [P15]. Another reported that the pace of new questions being added (around one every two days) was ‘too quick’ and done ‘before a number of people had a chance to answer‘. One participant felt **Facilitators sometimes did not accurately summarise participants’ comments,** and that some **participants might feel reluctant to correct a Facilitator** [P3].

	Facilitators reported finding it a challenge to separate 'personal' experiences when facilitating. One Facilitator noted 'Separating out my parent role and Facilitator role has been hard. Harder than I thought it would be'. One Facilitator also noted 'The discussion was too rapid. The group needed more time to work through the process of the online conversation. It felt too rushed.'
Views on which stage of the research people should be involved:<br>**3 participants** views on what the outcome or output of the involvement could be	One participant noted that **involving people in the design stage is more practical** as once research begins 'you can't change direction or question' [P3]. Two participants stated that **identifying topics and research development were appropriate stages for people to be involved**. One participant noted that **people 'must influence the research agenda including the questions, how they are funded, research design, data analysis, interpretation and dissemination of results'** [P16]
Who should the data from this project be shared with?:<br><br>**0 participants** shared views about who data from this project shared with	
Views on what the outcome or output of the involvement could be:<br><br>**0 participants** shared views about what the outcome or output of the involvement could be	

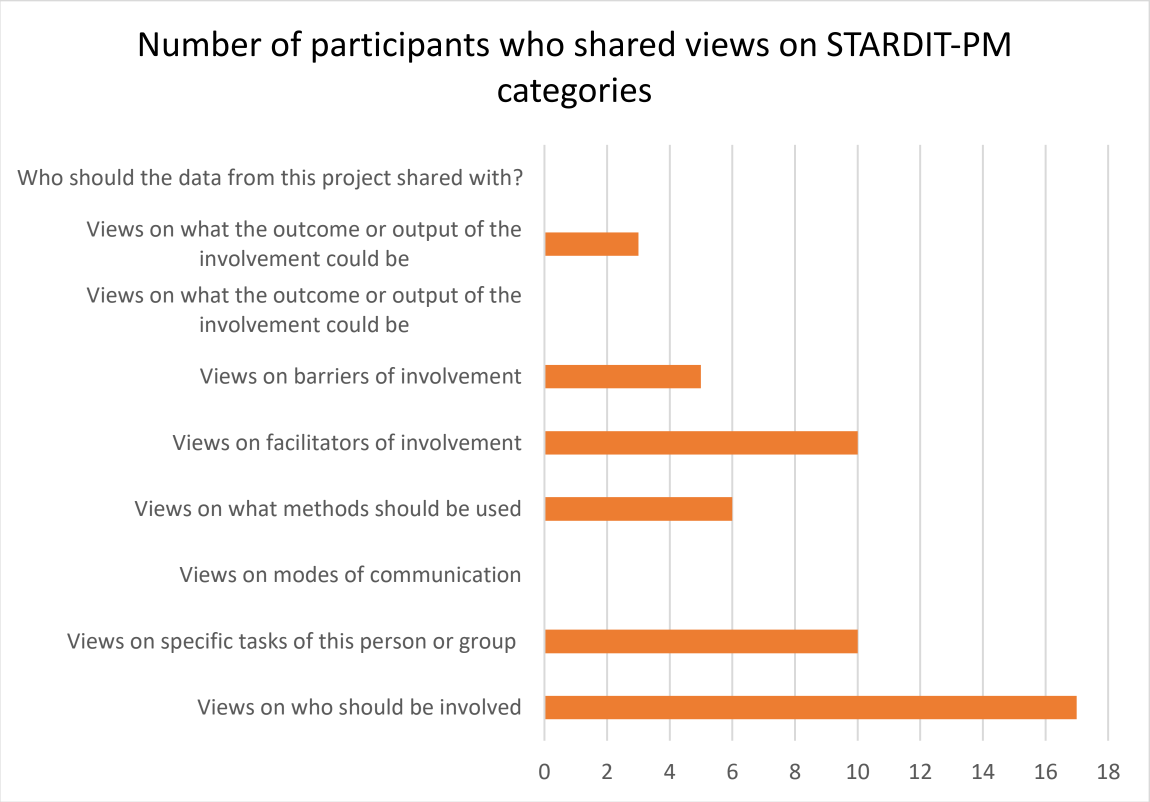

Data

This table summarises all the data sources used for the case study.

Data Category	Data point description
Diary	AusEE research diary of lead investigator (JN) – including reflections
Emails and meeting notes	Email, meeting notes and Notes from planning and discussion. This included involvement of participant representatives in co-refining study design.
Online pre-discussion survey	Informed consent and pre-discussion survey data
Learning resources for participants and Facilitators	Learning resources giving information about genomics and using Loomio
Online discussion with participants	Text data from online Loomio discussion with participants
Online discussion with Facilitators	Text data from online Loomio discussion between Facilitators of two parallel studies
Online post-discussion survey	Post-discussion survey data from participants
Follow up survey for Facilitators	Post-discussion survey data from Facilitators

Demographic information

Category	Pre-discussion survey	Post discussion Survey
**Gender**		
Female	28	8
Male	1	0
**Age**		
20-24 years	1	0
25-29 years	1	0
30-34 years	3	1
35-39 years	12	0
40-44 years	7	2
45-49 years	3	1
50-54 years	3	1
**Educational background ('highest' only counted)**		
Prefer not to say	1	0
I have qualifications or professional experience in genomics	1	0
Degree (bachelors), diploma or post-graduate ('higher')	26	6
High school qualifications (ages 16-19)	2	1
Middle school qualifications (up to age 16) ('lower')	1	0
**How would you describe yourself?**		
A carer of a person with EGID	1	1
A parent of a person with EGID	22	5
A partner, family member or loved one of a person with EGID	1	0
A person with EGID	5	1
A person with EGID and a parent of a person with EGID	1	0
**In which country do you live (or spend most time)?**		
Australia	28	6
New Zealand	1	0

Widening and narrowing for each question asked in pre and post survey

**Who should influence which aspects of research?**	**Change to wider**	**No change**	**Change to narrower**
**Finding questions to ask**	3	1	1
**Deciding which questions to prioritize and fund**	3	1	1
**Deciding how to try and answer the question** (the research method)	3	2	0
**Attempting to answer the question (carrying out the research, including collecting information)**	2	2	1
**Trying to understand if it is possible to the answer the question** (analysing the information)	2	3	0
**Sharing the information that has been found, and any answers that may have emerged** (dissemination and publication)	3	2	0
**Ensuring that any information or answers are able to be used to help people in practice, policy or future research** (sometimes called research translation)	4	1	0
**Deciding if the way of asking the question and all the other stages of the research were appropriate** (evaluating the research method and any impacts)	2	1	0
**Designing how people are involved in the research**	2	1	0
**Change totals**	**24**	**14**	**3**

Participant experience

How would you rate your overall experience of participating in the online discussion	
Excellent	9
Somewhat good	6

How would you rate how we conducted the survey and discussion format	
Excellent	11
Somewhat good	3
Neither good nor bad	1

How would you rate the support you received to be involved (for example, practical support such as instructions for using the online tools)?	
Excellent	11
Somewhat good	4

How would you rate the information and learning materials you were given before the event	
Excellent	11
Somewhat good	5
Neither good nor bad	1

Did you feel you meaningfully contributed to the discussion?	
Yes	8
Unsure	7

Learning resources

A number of different learning resources were shared with participants at different stages of the process. This included a short 60 second online video about the study, giving information about the context and purpose[2], a one page infographic summary of a scoping review about genomics research[3], and a short two-page summary of genomics and contemporary research relating to EoE was co-created with AusEE, the study team and experts in genomics[4].

Learning resources were both co-created and selected by the investigator team, working in partnership with the Australian Genomics Health Alliance and co-refining the selection with potential participants. In addition, in order to support the Enablers in providing good quality information, a number of were curated into a list to be available for Enablers to share during the online discussion, if they became relevant to aspects of the discussion in order to help inform people. The table below provides a summary of which learning resources were shared at which stage of the process.

Summary of learning resources

Stage	Title	Media	Summary	Authorship
**Stage 2: Before consent**	What is genomic testing?	Portable Document Format (PDF)	Simple infographic explaining the basics of genomics research in plain English	Australian Genomics Health Alliance[5]
	What is genomics?	Online video animation with audio narration and subtitles	A 6 minute video outlining the principles of genomics research	Genome BC[6]
**Stage 3: Before online discussion**	Definitions and explanations	PDF	These definitions and explanations were used as a glossary to explain the main concepts of this research project.	Investigator team
	Genomics and involvement	Online video with hard-coded text	A 2 minute video exploring why people should be involved in genomics	Jack Nunn[2]
	Infographic summary of scoping review	PDF (infographic – images and text)	A one page summary of the main findings from a recent scoping review about involving people in genomics[7]	Jack Nunn et al[3]
	A summary of EGID and relevant genomics research	PDF (text with hyperlinks)	A co-created learning resource updating people on what is known so far, what research is currently being done and what might be the future	Jack Nunn et al[4]
	Guide to using Loomio	PDF (text with hyperlinks)	A co-created learning resource giving practical advice for using the online discussion platform Loomio	Investigator team
**Additional resources available to facilitator**	Inheriting genomic conditions (chapter)	Webpage	An additional resource if participants wanted more information about inherited conditions	U.S National Library of Medicine[8]

This document contains additional data relevant to the case study 'Involving people affected by a rare condition in shaping future genomic research'. Contact Jack.Nunn@Latrobe.edu.au - orcid.org/0000-0003-0316-3254

	Data in the 100,000 Genomes Project	Online video animation with voice over and subtitles	An example of the bioinformatic pathway (specific to Genomics England) but generalisable (talks about access review committee)	Genomics England[9]
	Ethical issues in human genomics and genomics	PDF (text with hyperlinks)	Additional resource for a relevant ethics discussion from a medical perspective	Centre for Genomics Education[10]
	Genes, DNA and cancer	Webpage (text with hyperlinks)	Good plain English information about genes in relation to cancer.	Cancer Research UK[11]
	How to Share Genomic Test Results With Family	Webpage (text with hyperlinks)	Good information about sharing genomic test results with family.	American Society of Clinical Oncology[12]
	How do you sequence a human genome?	Image file (infographic – images and text)	Infographic about the stages of genome sequencing	Genomics England[13]

References

1. Nunn JS, Shafee T, Chang S, et al. Standardised Data on Initiatives - STARDIT: Alpha Version. 2019. doi:10.31219/OSF.IO/5Q47H
2. Jack Nunn. Genomics Research and Involving People. La Trobe University Library. https://web.archive.org/web/20200306053056/https://www.youtube.com/watch?v=21TmEfErDcU. Published 2018. Accessed March 6, 2020.
3. Nunn JS. Public involvement activities in 96 global genomics projects. 2018. https://doi.org/10.26181/5b63c24cc1b16.
4. Jack Nunn. Eosinophilic Gastrointestinal Disorders and genomics. https://web.archive.org/web/20200306051645/https://www.ausee.org/EoE and genomics.pdf. Published 2018. Accessed March 6, 2020.
5. Australian Genomics Health Alliance. What is genomic testing? https://web.archive.org/web/20200306050554/https://www.genomicsinfo.org.au/wp-content/uploads/2019/02/What-is-genomic-testing-v9.pdf. Published 2019. Accessed March 6, 2020.
6. Genome BC. What is Genomics? https://web.archive.org/web/20191221110313/https://www.youtube.com/watch?v=mmgIClg0Y1k. Published 2010. Accessed March 6, 2020.
7. Nunn JS, Tiller J, Fransquet PD, Lacaze P. Public Involvement in Global Genomics Research: A Scoping Review. *Front Public Heal*. 2019;7:79. doi:10.3389/FPUBH.2019.00079
8. U.S National Library of Medicine. What does it mean if a disorder seems to run in my family? https://web.archive.org/web/20180529060700/https://ghr.nlm.nih.gov/primer/inheritance/runsinfamily. Published 2018. Accessed March 6, 2020.
9. Genomics England. Data in the 100,000 Genomes Project (version 1.0 02/09/15) . https://web.archive.org/web/20200306055926/https://m.youtube.com/watch?v=nneWFaJ6Hfc. Published 2015. Accessed March 6, 2020.
10. Centre for Genetics Education. Ethical issues in human genetics and genomics. https://web.archive.org/web/20200306060134/https://www.genetics.edu.au/publications-and-resources/facts-sheets/fact-sheet-19-ethical-issues-in-human-genetics-and-genomics. Published 2018. Accessed March 6, 2020.
11. Cancer Research UK. Genes, DNA and cancer . https://web.archive.org/web/20200306060347/https://www.cancerresearchuk.org/about-cancer/what-is-cancer/genes-dna-and-cancer. Published 2017. Accessed March 6, 2020.
12. American Society of Clinical Oncology. How to Share Genetic Test Results With Family . https://web.archive.org/web/20200306060636/https://www.cancer.net/blog/2017-03/how-share-genetic-test-results-with-family. Published 2017. Accessed March 6, 2020.
13. Genomics England. How do you sequence a human genome? https://web.archive.org/web/20200306060635/https://www.genomicsengland.co.uk/wp-content/uploads/2015/10/Seq-infographic-FNL-061015-01-01HI-RES-01.jpg. Published 2015. Accessed March 6, 2020.
