# Supplementary material for: Involving people affected by a rare condition in shaping future genomic research
Source: Res Involv Engagem. 2021 Mar 15;7:14. doi: 10.1186/s40900-021-00256-3 (PMC7958104; doi:10.1186/s40900-021-00256-3)
Supplement: Supplementary file 2 — Additional file 2. STARDIT report. [file 40900_2021_256_MOESM2_ESM.pdf]

Standardised Data on Initiatives (STARDIT) report – Alpha Version

About this report

This report uses the Standardised Data on Initiatives Alpha version (STARDIT)[1].

STARDIT Report Alpha Version

Identifying information	
Initiative name	Involving people affected by a rare genetic disorder in shaping future research
Geographic location or scope	Australia
Date range (planned start and end dates of initiative)	2017-2020
Purpose of the initiative	Participatory action research to involve people affected by a rare disease in shaping future research, by using online discussions.
Organisations or other initiatives involved (list all if multi-centre)	1. School of Psychology and Public Health, La Trobe University
Funding sources	School of Psychology and Public Health, La Trobe University
Clinical trial registration details (if applicable)	N/A
Ethics approval (if applicable)	The La Trobe University Human Ethics Committee approved this study.<br>Project number: HEC18242<br>Project Title: Genomics Research and Involving People: AusEE
Other relevant information (free text)	This report describes involving people affected by a rare disease in shaping future research, by using online discussions exploring how they would like to be involved in future research.
At which stage of the research project has this report been written?	**After** the participatory action research occurred.
Methods of the initiative (what is planned to be done, or is being reported as done)	The research process was co-designed using a participatory action research method to involve people affected by a rare disease in the co-design of online discussions to explore future genomic research with members of the group.
**Report authorship**	
Name	Jack Nunn
Public domain profiles, institutional pages	https://scholars.latrobe.edu.au/display/j2nunn
Open Researcher and Contributor ID (orcid.org)	https://orcid.org/0000-0003-0316-3254

Tasks in report completion	Main author
Date of report authorship	24th July 2020
Key contact at initiative for confirming report content (include institutional email address)	Jack Nunn, PhD researcher, School of Psychology and Public Health, La Trobe University, jack.nunn@latrobe.edu
**Involvement**	
Who was involved or how would you label groupings of those involved	Group 1: Academic research investigators (Jack Nunn and Paul Lacaze)<br>Group 2: People affected by the rare disease representing the charity AusEE with experience of academic research (Kylie Gwynne)<br>Group 3: People affected by the rare disease representing the charity AusEE (Sarah Gray)<br>Group 4: People affected by the rare disease who are members of the online community and participated in the study
How many people were in each grouping label?	Group 1: 2<br>Group 2: 1<br>Group 3: 1<br>Group 4: 25
Specific tasks of this person or group (list as many as possible) – *including any information about why certain people were included or excluded in certain tasks*	Group 1 and 2: Involved in co-designing every stage of the process, analysing data and member checking during the thematic analysis<br>Group 3: Involved in co-designing the recruitment and giving feedback on the proposed study design and as an author of the paper.<br>Group 4: Invited to give feedback on the paper
How were these people involved (what methods were used)	Group 1 - 3: Face to face meetings, video calls, email communication, shared online documents, teleconferences.<br>Group 4: Invited to give feedback on paper and be acknowledged for contribution
Enablers of involvement (what do you expect will help these people get involved – or what helped them get involved)	Giving people time to read resources. Clear communication about the intention of involving people. Have multiple modes of communication for involving people.
Barriers of involvement (what do you expect will inhibit these people from getting involved – or what inhibited them from getting involved). Are there any known equity issues which may contribute?	Face-to-face meetings were difficult to organise. The study team were located in different states of Australia. Unclear communication about intentions and purpose of the involvement contributed to confusion (explaining how involvement is distinct from participation was

	challenging). Ensuring those involved had enough time to give feedback was also a challenge.
What was the outcome or output of the involvement of these people? What changed as a result of involving people?	Improved participant information resources, improved wording that was culturally appropriate (using terminology preferred by the group to describe themselves), improved online discussion, improved learning resources for participants, improved co-design process.
Which stage of the initiative were these people involved? (select from list of pre-defined stages or allow 'other')	Group 1 and 2: All stages<br>Group 3: Co-design, evaluation, dissemination<br>Group 4: evaluation, dissemination
What was the estimated financial cost for involving people. How much time did it take. Were there any costs that cannot be measured financially?	$0 AUD – people volunteered their time.<br>The total number of hours volunteered (excluding participation) is estimated to be 75.
What worked well, what could have been improved? Was anything learned from the process of involving these people?	The co-design process took longer than expected owing to ethical 'grey areas' with no clear instruction on whether ethics approval was required to involve people in co-design. As a result an ethics application was made and subsequent feedback from the co-design process was integrated using modifications to the ethics application.<br><br>Involving potential participants in co-defining language used to describe the group of people affected helped ensure that language was acceptable and appropriate.
**Mapping financial or other 'interests'**	
Describe any financial relationship or other interest this person has to this project	Two investigators are affected by the rare disease, which is why they were invited to be part of the study
Describe any conflicting or competing interests	N/A
**Data**	
Who is the data from this intervention shared with?	It will be published open access in peer reviewed journals with identifying information removed in order to anonymise it as much as possible.
How is it stored and hosted?	It will be shared on a public domain repository.
Who is analysing the data?	Group 1-3: The study team described above<br>Group 4: participants were invited to review the analysis and give feedback to ensure they felt it reflected their experience of the process

What methods will be used to analyse the data (including a link to any relevant code and information about validity)	We used case study methodology to describe our experience involving participants in the co-design of the proposed study. We collected and analysed both qualitative and quantitative data during the involvement activities.<br><br>We analysed data from online surveys and online discussions with participants. In addition, data from the study team communications was included, such as meeting notes, emails, reflexive diary entries and survey responses of study investigators. Coding and thematic analysis of qualitative data was carried out by two authors independently and checked by other authors.
How is information about this data disseminated?	1. It will be published in an open access journal<br>2. It will be shared with participants of the research and also other members of the sibling group who have joined it since the study commenced<br>3. Learning from this process will be presented at conferences, shared on social media and through other channels (such as podcasts).
Who 'owns' the data or claims any kind of 'intellectual property' (include relevant licensing information)	Confidential data collected as part of the study is stored according to laws and the data access plan approved by La Trobe University.<br><br>The authors maintain 'ownership' of the data in the paper and is shared under the Creative Commons license used by the publishing journal.
Who controls access to the data	The study team, La Trobe University and participants will be involved in any future data access decisions.
How is/will the data be 'Findable, Accessible, Interoperable, Reusable' according to the FAIR criteria?	Data will be shared in the public domain and licensed under a Creative Commons license.
**Impacts and outcomes**	
What new knowledge has been generated? (if appropriate, include effect size, relevant statistics and level or evidence)	1. Involving participants in co-designing the research process resulted in a number of changes to the study design, including improving language used in recruitment and learning resources<br>2. The process of involving people can be viewed as a learning experience for both

	the participants involved and study team members. The process changed participants' views about who should be involved, which can be viewed as an impact of 'transformative learning'.
What was learned	Involving people in online discussions about involvement in research changes people's views about who should be involved in research, including participants 'widening' their views about who should be involved in research to include more people.
Knowledge translation	1. Knowledge from this process will inform the design of a future genomic research<br>2. Learning from this process can inform future involvement activities
Outcomes	Learning from this process informed subsequent discussions in the charity AusEE about involvement in research, including proposed improved ways of involving people
How has or how will this be measured?	Future STARDIT reports
Who is involved in measuring this?	The study team and participants

References

1. Nunn JS, Shafee T, Chang S, et al. Standardised Data on Initiatives - STARDIT: Alpha Version. 2019. doi:10.31219/OSF.IO/5Q47H
