# Supplementary material for: Involving people affected by a rare condition in shaping future genomic research
Source: Res Involv Engagem. 2021 Mar 15;7:14. doi: 10.1186/s40900-021-00256-3 (PMC7958104; doi:10.1186/s40900-021-00256-3)
Supplement: Supplementary file 3 — Additional file 3. GRIPP2 report. [file 40900_2021_256_MOESM3_ESM.pdf]

GRIPP2 report for ‘Involving people affected by a rare condition in shaping future genomic research’

This report has been completed using the ‘GRIPP2 reporting checklists: tools to improve reporting of patient and public involvement in research’ available at https://doi.org/10.1136/bmj.j3453.

GRIPP2 short form

Section and topic	Category description	Data
1: Aim	Report the aim of PPI in the study	Participatory action research to involve people affected by a rare disease in shaping future research, by using online discussions.
2: Methods	Provide a clear description of the methods used for PPI in the study	The research process was co-designed using a participatory action research method to involve people affected by a rare disease in the co-design of online discussions to explore future genomic research with members of the group. Participants were also involved in analysing the data and checking the final version of the paper.
3: Study results	Outcomes—Report the results of PPI in the study, including both positive and negative outcomes	The input of the representatives during the planning and co-design stage had clear positive impacts, particularly in improving educational resources and ensuring the online discussion was advertised using wording appropriate to the existing online community. For example, representatives from AusEE helped change the study design to include explicit opportunities for participants to learn more about genomics and EoE, avoiding participation being perceived as having a one-way benefit. During the co-design process it was also decided to exclude people who were under 18 and people who stated they were representing someone who was over 18, as people who were 18 and over had the choice to represent themselves.

Section and topic	Category description	Data
		Enablers of involvement: Giving people time to read resources. Clear communication about the intention of involving people. Have multiple modes of communication for involving people.<br><br>Barriers of involvement: Face-to-face meetings were difficult to organise. The study team were located in different states of Australia. Unclear communication about intentions and purpose of the involvement contributed to confusion (explaining how involvement is distinct from participation was challenging). Ensuring those involved had enough time to give feedback was also a challenge.
4: Discussion and conclusions	Outcomes—Comment on the extent to which PPI influenced the study overall. Describe positive and negative effects	Involvement improved participant information resources, improved wording that was culturally appropriate (using terminology preferred by the group to describe themselves), improved online discussion, improved learning resources for participants, improved co-design process.<br><br>Involving potential participants in co-defining language used to describe the group of people affected helped ensure that language was acceptable and appropriate.<br><br>Involving participants in co-designing the research process resulted in a number of changes to the study design, including improving language used in recruitment and learning resources.<br><br>The process of involving people can be viewed as a learning experience for both the participants involved and study team members. The process changed participants' views about who should be involved, which can be viewed as an impact of 'transformative learning'.
5: Reflections/critical perspective	Comment critically on the study, reflecting on the things that went well and	The co-design process took longer than expected owing to ethical 'grey areas' with no clear instruction on whether ethics approval was required to involve people in

Section and topic	Category description	Data
	those that did not, so others can learn from this experience	co-design. As a result an ethics application was made and subsequent feedback from the co-design process was integrated using modifications to the ethics application.<br><br>Involving potential participants in co-defining language used to describe the group of people affected helped ensure that language was acceptable and appropriate.<br><br>Involving participants in co-designing the research process resulted in a number of changes to the study design, including improving language used in recruitment and learning resources.<br><br>The process of involving people can be viewed as a learning experience for both the participants involved and study team members. The process changed participants' views about who should be involved, which can be viewed as an impact of 'transformative learning'.<br><br>Involving people in online discussions about involvement in research changes people's views about who should be involved in research, including participants 'widening' their views about who should be involved in research to include more people.
